# Supplementary material for: The impact of extended reality cognitive behavioral therapy on mental disorders among children and youth: A systematic review and meta-analysis protocol
Source: PLoS One. 2025 Mar 6;20(3):e0315313. doi: 10.1371/journal.pone.0315313 (PMC11884679; doi:10.1371/journal.pone.0315313)
Supplement: S2 Fig — (PDF) [file pone.0315313.s002.pdf]

(“Virtual Reality Cognitive Behavioral Therapy” OR “Virtual Reality Cognitive Behavioural Therapy” OR "Virtual Reality CBT" OR "VRCBT" OR "Virtual Reality Exposure Therapy" OR "VRET" OR "Virtual reality training" OR “cognitive behavior* therapy with virtual reality” OR “Augmented Reality with Cognitive Behavioral Therapy” OR “Augmented Reality with Cognitive Behavioural Therapy” OR“AR-CBT” OR “ARET” OR “Mixed Reality with Cognitive Behavioral Therapy” OR “Mixed Reality with Cognitive Behavioural Therapy” OR “MR-CBT” OR “extended reality with cognitive behavioral therapy” OR “extended reality with cognitive behavioural therapy” OR “XR-CBT” OR “XRET”) AND ("Mental Health" OR Psychological Wellbeing OR Wellbeing OR Anxiet* OR Phobia OR OCD OR Depress* OR PTSD OR Disorder* OR Behaviour* OR Behavior OR Psycholog*) AND (Youth OR Adolescent* OR Teen* OR Student* OR Child* OR college student*)

S2 Fig. Search strategy to be used for all databases (PubMed, Embase, PsycINFO, Web of Science, and Scopus)
