# Supplementary material for: The impact of extended reality cognitive behavioral therapy on mental disorders among children and youth: A systematic review and meta-analysis protocol
Source: PLoS One. 2025 Mar 6;20(3):e0315313. doi: 10.1371/journal.pone.0315313 (PMC11884679; doi:10.1371/journal.pone.0315313)
Supplement: Attachment — Submitted filename: PONE-D-24-35309_updated.pdf [file pone.0315313.s003.pdf]

Manuscript Click here to access/download;Manuscript;XR-CBT protocol main text.docx

The impact of extended reality cognitive behavioral therapy on mental health outcomes among children and youth: a systematic review protocol

Madeline Li [a], Tarun Reddy Katapally [a,b,c] *

[a]DEPtH Lab, School of Health Studies, Faculty of Health Sciences, Western University, London, Ontario, Canada N6A 5B9

[b]Department of Epidemiology and Biostatistics, Schulich School of Medicine and Dentistry, Western University, London, Ontario, Canada N6A 3K7

[c]Children's Health Research Institute, Lawson Health Research Institute, 750 Base Line Road East, Suite 300, London, Ontario, Canada N6C 2R5

*** Corresponding author**

Tarun Reddy Katapally

E-mail: tarun.katapally@uwo.ca (TRK)

Madeline Li contributed to conceptualization, investigation, visualization, and writing – original draft preparation, review and editing

Tarun Reddy Katapally contributed to conceptualization, investigation, funding acquisition, supervision, and writing – review and editing

Abstract

The prevalence of mental health issues among children and youth have significantly increased, with rising rates of anxiety, depression, and other psychological disorders globally. Despite the widespread adoption of cognitive behavioral therapy (CBT) as a standardized treatment for various mental health issues, its efficacy can be constrained due to limited patient engagement, lack of commitment, and mental health stigma, all challenges pronounced among children and youth. In this context, extended reality (XR) technologies (including virtual reality, augmented reality, and mixed reality) have emerged as innovative therapeutic tools offering immersive and engaging environments to overcome the limitations of traditional CBT. Currently, no systematic reviews have examined the impacts of extended reality cognitive behavioral therapy (XR-CBT) on mental health outcomes within the child and youth demographic. Thus, this systematic review protocol aims to evaluate the impact of XR-CBT on mental health outcomes among children and youth. A comprehensive literature search was conducted using five electronic databases: PsycINFO, PubMed, EMBASE, Scopus, and Web of Science. The search strategy will include terms related to virtual reality, augmented reality, mixed reality, cognitive behavioral therapy, exposure therapy, youth, children, and mental health. Two independent reviewers will evaluate the search results from the five selected databases based on the pre-determined search strategy. Findings from this study will provide insights regarding the potential of XR-CBT to enhance mental health outcomes, which can contribute to addressing the escalating mental health crisis among children and youth.

1. Introduction

Mental illness constitutes a significant public health challenge, particularly among youth where its prevalence is notably high. This demographic is increasingly susceptible to conditions, with studies indicating that 1 in 7 adolescents experience a mental health disorder in any given year (1), profoundly affecting their development (2), academic performance (3), and overall quality of life (2). Moreover, mental health issues that originate in youth often extend into adulthood, with approximately 50% of all lifetime mental health conditions have been shown to begin by the age of 14 (4). Furthermore, the global economic burden of mental health issues among youth is substantial, amounting to $1 trillion annually (5). This underscores the need for innovative therapeutic interventions to address the unique mental health challenges faced by youth.

An established therapeutic approach for treating various mental health conditions is cognitive behavioral therapy (CBT). CBT focuses on identifying and challenging negative thought patterns and behaviors and modifying maladaptive behavioral issues (6). CBT encompasses different subtypes of therapeutic interventions, including exposure therapy (7), trauma-focused CBT (8), and behavioral activation therapy (9), which can be effective in treating anxiety disorders (10), phobias (11), depression (12), and post-traumatic stress disorders (PTSD) (11). Despite being a gold standard therapeutic intervention (13), CBT does have several limitations. It requires active participation (14) and a high level of commitment from patients (14), which can be challenging for youth. Furthermore, the traditional face-to-face format may not engage younger patients effectively. For some types of phobias or anxieties, it can be difficult to provide the necessary exposures in a controlled and practical manner during therapy sessions (15). Moreover, seeking traditional face-to-face therapies such as CBT often carry a stigma, especially among children and youth (16), who may be reluctant in receiving mental health care due to societal perceptions and

judgement from peers and family (17–19). These limitations highlight the need for more adaptable and engaging therapeutic approaches to effectively address mental health issues among youth and children.

A promising solution to these limitations is the use of extended reality cognitive behavioral therapy (XR-CBT). This innovative intervention employs advanced technologies, including virtual reality (VR), augmented reality (AR), or mixed reality (MR) (20), to provide patients with a controlled and safe virtual environment to confront and manage their mental health challenges (21). XR-CBT serves as an umbrella term encompassing various specific approaches, with virtual reality cognitive behavioral therapy (VR-CBT) (15,22,23) and virtual reality exposure therapy (VRET) (24–26) being the most utilized methodologies. These specialized forms of therapy incorporate the immersive nature of VR to create realistic scenarios where patients can practice coping strategies, desensitize themselves to anxiety-provoking stimuli, and receive real-time feedback from their therapists (27–30). By integrating these advanced technologies, XR-CBT has the potential to enhance the traditional CBT framework, making therapeutic interventions more engaging and effective for youth patients. Beyond enhancing the traditional CBT framework, XR-CBT holds potential for integration into digital mental health platforms (31). As virtual care continues to expand (32,33), incorporating XR-CBT could align with this growing trend, offering new possibilities for child and youth mental health interventions. Such integration can provide another effective alternative to traditional face-to-face CBT therapy, which is crucial for the consideration of XR-CBT in the future of mental health care.

Considering the high prevalence of youth mental health disorders (1,2,4) and the limitations of traditional CBT (14–19), it is crucial to investigate the efficacy of XR-CBT for this demographic. Currently, no systematic reviews have explored the impact of XR-CBT on mental health outcomes

in children and youth. Prior reviews have examined XR-CBT therapy interventions; however, they have primarily focused on general populations rather than children and youth (24,34,35). While reviews on XR interventions among children and youth exist, they have focused on specific mental health issues, such as anxiety (36) or schizophrenia (37), rather than evaluating the broader applications of XR-CBT. Thus, the objective of this review is to provide a comprehensive overview of the impacts of XR-CBT interventions on mental health outcomes among youth and child populations.

This review aims to:

1. Evaluate the efficacy of XR-CBT interventions in treating mental health symptoms in children and youth;
2. Explore the customization and personalization of XR-CBT interventions;
3. Investigate the potential for integration of XR-CBT into digital mental health platforms;
4. Develop recommendations for future development, implementation, and evaluation of XR-CBT that aims to treat youth and child mental health.

2. Methods

2.1 Design

This systematic review protocol follows the Preferred Reporting Items for Systematic Review and Meta-Analysis Protocols (PRISMA-P) 2020 guidelines **(Figure S1).**

2.1.1 Research question

In this systematic review, the main research question that will be examined is “what is the impact of XR-CBT on mental health outcomes among children and youth?”. Studies will focus on the use of XR-CBT in treating mental illnesses among childre[illegible] youth, encompassing technologies such as VR-CBT, augmented reality cognitive behavioral therapy (AR-CBT), and mixed reality cognitive behavioral therapy (MR-CBT). Mental health outcomes will be analyzed in this study, specifically looking for whether a change in mental health symptoms occurs after the implementation of XR-CBT interventions.

2.2.2 Eligibility criteria

This systematic review will include studies investigating the effects of XR-CBT interventions on mental health symptoms in children and youth aged 25 years and younger. Participants with any existing mental illness or disorder will be included. Studies that only explore participants' experiences or understanding of XR-CBT interventions, and those that do not specifically assess the effects of XR-CBT on mental health outcomes (i.e., feasibility studies, developmental studies) will be excluded. Moreover, case studies will be excluded, as this systematic review will assess the efficacy of XR-CBT interventions using studies that provide higher levels of evidence. Additionally, this review will only examine studies published in English. In consideration of ongoing technological advancements, this review aims to present a comprehensive overview of the latest applications of XR-CBT by analyzing studies published within the past decade, specifically from January 2014 to June 2024. **Table 1** presents a detailed overview of the inclusion and exclusion criteria of this systematic review.

Table 1: Inclusion and exclusion criteria for this systematic review

Concept	Inclusion	Exclusion
Age	Ages 1-25 years	All other ages
Therapeutic intervention	VR-CBT, VRET, AR-CBT, ARET, MR-CBT, XR-CBT, XRET	Therapeutic interventions that do not integrate XR-CBT
Mental health outcomes	Indicators of mental wellbeing such as changes mental health symptoms	Physical disorders/other health issues not relating to mental health, economic, cognitive, and physical outcomes not related to mental health
Language	English	All other languages
Publication status	Peer-reviewed literature	Grey literature
Date of publication	January 2014 to June 2024	All other dates
Type of study	Original articles	Review articles, feasibility studies, developmental studies, case studies

2.3 Study Records

2.3.1 Data sources

The study will use the following databases to analyze key studies on this topic: Embase, PubMed, Web of Science, PsycInfo, and Scopus. Embase and PubMed will be used as a source for biomedical research, providing valuable studies on clinical research on mental health disorders and medical devices. PsycInfo will be used to gain comprehensive coverage of psychology and behavioral science literature. As this systematic review includes components of psychology, medicine, and technology, Scopus and Web of Science will be used to provide a broad and

inclusive perspective from various fields. The advanced search tool will be utilized for all databases to ensure precise and comprehensive retrieval of relevant studies.

2.3.2 Search strategy

In consultation with domain-specific librarians to ensure credibility, four principal thematic categories were incorporated into the search strategy: therapeutic intervention, mental health outcome, and age. A key concepts map is provided in **Table 2**. Moreover, the reference list of the peer-reviewed literature included will also be manually examined to identify any relevant articles that might have been missed by the initial search criteria.

Table 2: Key concepts map

**Thematic category**	**Search terms**
Therapeutic intervention	Virtual Reality Cognitive Behavioral Therapy, Virtual Reality Cognitive Behavioral Therapy, Virtual Reality CBT, VRCBT, Virtual Reality Exposure Therapy, VRET, Virtual reality training, cognitive behavior* therapy with virtual reality, Augmented Reality with Cognitive Behavioral Therapy, Augmented Reality with Cognitive Behavioral Therapy, AR-CBT, ARET, Mixed Reality with Cognitive Behavioral Therapy, Mixed Reality with Cognitive Behavioral Therapy, MR-CBT, extended reality with cognitive behavioral therapy, extended reality with cognitive behavioral therapy, XR-CBT, XRET
Mental health outcome	Mental Health, Psychological Wellbeing, Wellbeing, Anxiet*, Phobia, OCD, Depress*, PTSD, Disorder*, Behaviour*, Behavior, Psycholog*
Age	Youth, Adolescent*, Teen*, Student*, Child*, college student*

2.3.3 Data extraction

After implementing the search strategy in each database, results will be transferred into Covidence software for systematic screening and management. In the initial stage, duplicates will be removed, and the remaining studies will be assessed for inclusion criteria based on their titles and abstracts by two independent reviewers, thus ensuring reliability. Any conflicts will be discussed and resolved between the two reviewers. In the next stage, the full texts of studies selected in the initial stage will be thoroughly reviewed by both independent reviewers. If any studies do not meet the inclusion criteria, they will be discussed between the reviewers before the data extraction process. Moreover, the Cochrane risk-of-bias tool will be used to assess for the risk of bias for individual studies included in the systematic review (38).

2.3.4 Data synthesis and analysis

Covidence will be utilized to present the results using the [illegible]SMA 2020 four-phase flow [illegible]am (39) and to systematically extract data from each of the included studies in the specified categories: year of publication, study design, aim of study, sample size of the study population, baseline [illegible]aracteristics of the study population, mental health outcomes, and type of therapeutic intervention. Moreover, assessment of XR-CBT interventions will be extracted, including customization and personalization, technology fea[illegible]s, description of XR environment, and any adverse outcomes.

3. Limitations

Participating in XR-CBT interventions requires access to advanced technologies, which may not be available to certain populations, particularly in low-income areas (40,41). This digital divide could lead to an underrepresentation of these groups in the included studies for this systematic

review. Additionally, another limitation of this systematic review is the potential language bias from including only English-language studies, which may exclude relevant research in other languages. Moreover, this study exclusively focuses on XR-CBT interventions and does not assess other XR therapies.

4. Conclusion

This systematic review will overview the existing evidence on the impacts of XR-CBT interventions on mental health outcomes among children and youth. Beyond assessing the efficacy of XR-CBT, this review will explore the customization and personalization of XR-CBT and provide actionable insights into the potential for XR-CBT to be integrated into digital mental health platforms. Additionally, findings from this review will not only contribute to the existing body of literature but also have the potential to inform the development of more effective, accessible, and engaging mental health interventions designed to meet the unique needs of youth. Given the significant increase of mental health illnesses among this demographic and the current constraints of traditional CBT, there is an urgent need for innovative and more effective therapeutic approaches. By advancing our understanding of XR-CBT, this research has the potential to initiate a shift in our current delivery of mental health care for children and youth, working to sustain long-term positive mental health outcomes.

Acknowledgements

We would like to thank the University Western Ontario librarians for their help in creating our literature search strategy.

References

1. Mental health of adolescents [Internet]. [cited 2024 Aug 16]. Available from: https://www.who.int/news-room/fact-sheets/detail/adolescent-mental-health

2. CDC. Centers for Disease Control and Prevention. 2024 [cited 2024 Aug 16]. What Is Children's Mental Health? Available from: https://www.cdc.gov/childrensmentalhealth/basics.html

3. Mitchell RJ, McMaugh A, Schniering C, Cameron CM, Lystad RP, Badgery-Parker T, et al. Mental disorders and their impact on school performance and high school completion by gender in Australia: A matched population-based cohort study. Aust N Z J Psychiatry. 2022 Dec 1;56(12):1602–16.

4. Mental Health Conditions [Internet]. NAMI. [cited 2024 Aug 16]. Available from: https://www.nami.org/about-mental-illness/mental-health-conditions/

5. The Lancet Global Health. Mental health matters. The Lancet Global Health. 2020 Nov 1;8(11):e1352.

6. Hofmann SG, Asnaani A, Vonk IJJ, Sawyer AT, Fang A. The Efficacy of Cognitive Behavioral Therapy: A Review of Meta-analyses. Cogn Ther Res. 2012 Oct 1;36(5):427–40.

7. nhs.uk [Internet]. 2021 [cited 2024 Aug 16]. How it works - Cognitive behavioural therapy (CBT). Available from: https://www.nhs.uk/mental-health/talking-therapies-medicine-treatments/talking-therapies-and-counselling/cognitive-behavioural-therapy-cbt/how-it-works/

8. Ramirez de Arellano MA, Lyman DR, Jobe-Shields L, George P, Dougherty RH, Daniels AS, et al. Trauma-Focused Cognitive Behavioral Therapy: Assessing the Evidence. Psychiatr Serv. 2014 May 1;65(5):591–602.

9. Uphoff E, Ekers D, Robertson L, Dawson S, Sanger E, South E, et al. Behavioural activation therapy for depression in adults - Uphoff, E - 2020 | Cochrane Library. [cited 2024 Aug 16]; Available from: https://www.cochranelibrary.com/cdsr/doi/10.1002/14651858.CD013305.pub2/full

10. Carpenter JK, Andrews LA, Witcraft SM, Powers MB, Smits JAJ, Hofmann SG. Cognitive behavioral therapy for anxiety and related disorders: A meta-analysis of randomized placebo-controlled trials. Depression and Anxiety. 2018;35(6):502–14.

11. Plaisted H, Waite P, Gordon K, Creswell C. Optimising Exposure for Children and Adolescents with Anxiety, OCD and PTSD: A Systematic Review. Clin Child Fam Psychol Rev. 2021 Jun 1;24(2):348–69.

12. Gautam M, Tripathi A, Deshmukh D, Gaur M. Cognitive Behavioral Therapy for Depression. Indian Journal of Psychiatry. 2020 Jan;62(Suppl 2):S223.

13. David D, Cristea I, Hofmann SG. Why Cognitive Behavioral Therapy Is the Current Gold Standard of Psychotherapy. Front Psychiatry [Internet]. 2018 Jan 29 [cited 2024 Aug 16];9.

Available from: https://www.frontiersin.org/journals/psychiatry/articles/10.3389/fpsyt.2018.00004/full

14. In brief: Cognitive behavioral therapy (CBT). In: InformedHealth.org [Internet] [Internet]. Institute for Quality and Efficiency in Health Care (IQWiG); 2022 [cited 2024 Aug 16]. Available from: https://www.ncbi.nlm.nih.gov/books/NBK279297/

15. Wu J, Sun Y, Zhang G, Zhou Z, Ren Z. Virtual Reality-Assisted Cognitive Behavioral Therapy for Anxiety Disorders: A Systematic Review and Meta-Analysis. Front Psychiatry [Internet]. 2021 Jul 23 [cited 2024 Aug 16];12. Available from: https://www.frontiersin.org/journals/psychiatry/articles/10.3389/fpsyt.2021.575094/full

16. Sheikhan NY, Henderson JL, Halsall T, Daley M, Brownell S, Shah J, et al. Stigma as a barrier to early intervention among youth seeking mental health services in Ontario, Canada: a qualitative study. BMC Health Serv Res. 2023 Jan 26;23:86.

17. Gulliver A, Griffiths KM, Christensen H. Perceived barriers and facilitators to mental health help-seeking in young people: a systematic review. BMC Psychiatry. 2010 Dec 30;10(1):113.

18. Radez J, Reardon T, Creswell C, Lawrence PJ, Evdoka-Burton G, Waite P. Why do children and adolescents (not) seek and access professional help for their mental health problems? A systematic review of quantitative and qualitative studies. Eur Child Adolesc Psychiatry. 2021 Feb 1;30(2):183–211.

19. Aguirre Velasco A, Cruz ISS, Billings J, Jimenez M, Rowe S. What are the barriers, facilitators and interventions targeting help-seeking behaviours for common mental health problems in adolescents? A systematic review. BMC Psychiatry. 2020 Jun 11;20(1):293.

20. Marr B. Forbes. [cited 2024 Aug 16]. What Is Extended Reality Technology? A Simple Explanation For Anyone. Available from: https://www.forbes.com/sites/bernardmarr/2019/08/12/what-is-extended-reality-technology-a-simple-explanation-for-anyone/

21. Pons P, Navas-Medrano S, Soler-Dominguez JL. Extended reality for mental health: Current trends and future challenges. Front Comput Sci [Internet]. 2022 Nov 18 [cited 2024 Aug 16];4. Available from: https://www.frontiersin.org/journals/computer-science/articles/10.3389/fcomp.2022.1034307/full

22. Seon Q, Mady N, Yang M, Karia M, Lashley M, Sescu C, et al. A Virtual Reality–Assisted Cognitive Behavioral Therapy for and With Inuit in Québec: Protocol for a Proof-of-Concept Randomized Controlled Trial. JMIR Res Protoc. 2023 May 24;12:e40236.

23. Loenen I van, Scholten W, Muntingh A, Smit J, Batelaan N. The Effectiveness of Virtual Reality Exposure–Based Cognitive Behavioral Therapy for Severe Anxiety Disorders, Obsessive-Compulsive Disorder, and Posttraumatic Stress Disorder: Meta-analysis. Journal of Medical Internet Research. 2022 Feb 10;24(2):e26736.

24. Freitas JRS, Velosa VHS, Abreu LTN, Jardim RL, Santos JAV, Peres B, et al. Virtual Reality Exposure Treatment in Phobias: a Systematic Review. Psychiatr Q. 2021 Dec 1;92(4):1685–710.

25. Rejbrand C, Fure B, Sonnby K. Stand-alone virtual reality exposure therapy as a treatment for social anxiety symptoms: a systematic review and meta-analysis. Upsala Journal of Medical Sciences [Internet]. 2023 Sep 15 [cited 2024 Aug 16];128. Available from: https://ujms.net/index.php/ujms/article/view/9289

26. Wechsler TF, Kümpers F, Mühlberger A. Inferiority or Even Superiority of Virtual Reality Exposure Therapy in Phobias?—A Systematic Review and Quantitative Meta-Analysis on Randomized Controlled Trials Specifically Comparing the Efficacy of Virtual Reality Exposure to Gold Standard in vivo Exposure in Agoraphobia, Specific Phobia, and Social Phobia. Front Psychol [Internet]. 2019 Sep 10 [cited 2024 Aug 16];10. Available from: https://www.frontiersin.org/journals/psychology/articles/10.3389/fpsyg.2019.01758/full

27. Riva G, Serino S. Virtual Reality in the Assessment, Understanding and Treatment of Mental Health Disorders. Journal of Clinical Medicine. 2020 Nov;9(11):3434.

28. Wiley E, Khattab S, Tang A. Examining the effect of virtual reality therapy on cognition post-stroke: a systematic review and meta-analysis. Disability and Rehabilitation: Assistive Technology. 2022 Jan 2;17(1):50–60.

29. Jeppesen UN, Due AS, Mariegaard L, Pinkham A, Vos M, Veling W, et al. Face Your Fears: Virtual reality-based cognitive behavioral therapy (VR-CBT) versus standard CBT for paranoid ideations in patients with schizophrenia spectrum disorders: a randomized clinical trial. Trials. 2022 Aug 15;23(1):658.

30. Gonçalves R, Pedrozo AL, Coutinho ESF, Figueira I, Ventura P. Efficacy of Virtual Reality Exposure Therapy in the Treatment of PTSD: A Systematic Review. PLoS One. 2012 Dec 27;7(12):e48469.

31. Ong T, Barrera JF, Sunkara C, Soni H, Ivanova J, Cummins MR, et al. Mental health providers are inexperienced but interested in telehealth-based virtual reality therapy: survey study. Front Virtual Real [Internet]. 2024 Jul 4 [cited 2024 Aug 16];5. Available from: https://www.frontiersin.org/journals/virtual-reality/articles/10.3389/frvir.2024.1332874/full

32. Shaver J. The State of Telehealth Before and After the COVID-19 Pandemic. Prim Care. 2022 Dec;49(4):517–30.

33. Bhatia RS, Chu C, Pang A, Tadrous M, Stamenova V, Cram P. Virtual care use before and during the COVID-19 pandemic: a repeated cross-sectional study. Canadian Medical Association Open Access Journal. 2021 Jan 1;9(1):E107–14.

34. Paul M, Bullock K, Bailenson J, Burns D. Examining the Efficacy of Extended Reality–Enhanced Behavioral Activation for Adults With Major Depressive Disorder: Randomized Controlled Trial. JMIR Mental Health. 2024 Apr 15;11(1):e52326.

35. Dhunnoo P, Wetzlmair LC, O'Carroll V. Extended Reality Therapies for Anxiety Disorders: A Systematic Review of Patients' and Healthcare Professionals' Perspectives. Sci. 2024 Jun;6(2):19.

36. Kothgassner OD, Felnhofer A. Lack of research on efficacy of virtual reality exposure therapy (VRET) for anxiety disorders in children and adolescents. Neuropsychiatr. 2021 Jun 1;35(2):68–75.

37. Dellazizzo L, Potvin S, Bahig S, Dumais A. Comprehensive review on virtual reality for the treatment of violence: implications for youth with schizophrenia. npj Schizophr. 2019 Jul 23;5(1):1–12.

38. RoB 2: A revised Cochrane risk-of-bias tool for randomized trials | Cochrane Bias [Internet]. [cited 2024 Aug 16]. Available from: https://methods.cochrane.org/bias/resources/rob-2-revised-cochrane-risk-bias-tool-randomized-trials

39. PRISMA statement [Internet]. [cited 2024 Aug 16]. PRISMA 2020 flow diagram. Available from: https://www.prisma-statement.org/prisma-2020-flow-diagram

40. Saeed SA, Masters RM. Disparities in Health Care and the Digital Divide. Curr Psychiatry Rep. 2021;23(9):61.

41. Sanders CK, Scanlon E. The Digital Divide Is a Human Rights Issue: Advancing Social Inclusion Through Social Work Advocacy. J Hum Rights Soc Work. 2021;6(2):130–43.

Click here to access/download
Supporting Information
PLOS ONE Figure (S1).docx
