# Supplementary material for: The impact of extended reality cognitive behavioral therapy on mental disorders among children and youth: A systematic review and meta-analysis protocol
Source: PLoS One. 2025 Mar 6;20(3):e0315313. doi: 10.1371/journal.pone.0315313 (PMC11884679; doi:10.1371/journal.pone.0315313)
Supplement: Attachment — Submitted filename: Reviewer responses october 2024.pdf [file pone.0315313.s006.pdf]

Reviewer #1:

The manuscript requires further proofreading. Minor grammatical and typographical errors affect readability. These need to be corrected to ensure the manuscript is clear and correct and meets PLOS ONE's standards for intelligibility. Ensure smooth transitions between sections and improve the overall flow.

Thank you for highlighting the need for further proofreading. We have carefully reviewed the manuscript for grammatical and typographical errors and ensured smoother transitions between sections to enhance the overall flow.

Clearly distinguish any exploratory aspects of the analysis. While the protocol outlines the methodology well, it would benefit from explicitly marking which parts of the analysis are exploratory to prevent potential flexibility in interpretation after the study is conducted.

Thank you for your comment. We have now clearly identified the exploratory aspects of our analysis in the revised manuscript. Specifically, the exploratory analyses will include: 1) Subgroup Analyses: subgroup analyses will be performed to examine differences by study design, intervention type, and participant characteristics; 2) Post-hoc exploratory meta-regression: In response to another reviewer, we will conduct a post-hoc exploratory meta-regression to assess the association between factors such as age, gender, time since diagnosis, and the severity of symptoms associated with mental disorders; 3) Sensitivity Analyses: We will conduct sensitivity analyses by excluding studies with small sample sizes or high risk of bias to assess the robustness of our primary findings. These exploratory analyses are now clearly distinguished with a subtitle "exploratory analyses" (line 243) from our other "pre-specified analyses" (line 221).

Double-check that all supplementary materials are fully included and correctly uploaded. This includes ensuring the full search strategy and supplementary data files are accessible and meet the journal's requirements.

Thank you for your helpful feedback. We carefully reviewed and confirmed that all supplementary materials are fully included and correctly uploaded. This includes ensuring the full search strategy for each database and supplementary data files are accessible and meet the journal's requirements. Supplementary files noted in the manuscript (**Figure S1** (line 137), and **Figure S2** (line 194)) are uploaded using the portal's instructions to ensure proper submission of the supplementary files.

So, please Conduct a thorough language revision, clarify exploratory analysis aspects, and ensure proper inclusion and access to all supplementary files.

Thank you for your valuable feedback. We have addressed your concerns as follows: 1) Language Revision: We have conducted a thorough revision of the manuscript to enhance clarity, improve readability, and ensure consistency throughout. We carefully reviewed the text to eliminate any ambiguities and improve the overall presentation of the study; 2) Exploratory Analyses: We have now explicitly clarified which parts of the analysis are exploratory. Specifically, the subgroup analyses, sensitivity analyses, and post-hoc exploratory meta-regression are designated as exploratory. These are clearly distinguished from the pre-specified analyses in the revised manuscript; 3) Supplementary Files: All supplementary files have been properly included and made accessible. We have ensured that they are correctly referenced in the manuscript and available for review to ensure transparency and full access to the data.

Reviewer #2

The addition to the introduction on XR therapies (lines 106-112) is strong. However, the authors might further highlight how this systematic review addresses gaps in previous research (perhaps adding a sentence or two) to emphasize its unique contribution.

Thank you for your feedback on the addition regarding XR therapies. In response to your suggestion, we have added two sentences to further highlight how this systematic review addresses gaps in previous research and emphasizes its unique contribution. Additionally, we have cited previous systematic reviews and meta-analyses to support this (lines 121-130):

"Currently, no systematic reviews have explored the impact of XR-CBT on mental disorders among children and youth. Prior reviews have examined the effectiveness of XR-CBT therapy interventions; however, they have primarily focused on general populations rather than children and youth (35,52,53). While reviews on XR interventions among children and youth exist, they have focused on specific mental disorders, such as anxiety (48) or schizophrenia (54), rather than evaluating the broader applications of XR-CBT. Notably, most XR reviews on mental disorders among children and youth have predominantly explored the VR dimension of XR (48–50,54–56), with limited focus on other XR interventions such as AR or MR. Furthermore, many systematic reviews have concentrated on other forms of XR therapies, such as XR distraction and relaxation therapy (57–59), rather than XR-CBT."

In the section discussing the use of a random-effects model for the meta-analysis (lines 213 to 220), it could be helpful to briefly explain why a random-effects model is chosen over a fixed-effects model.

Thank you for your comment. We have selected a random-effects model for the meta-analysis because it accounts for potential variations both between studies and among individuals. This model is particularly appropriate in the context of our review, as it adjusts for heterogeneity across studies, recognizing that the true effect size may differ due to variations in study design, populations, and interventions. In contrast to a fixed-effects model, which assumes a common effect size across all studies, the random-effects model provides a more appropriate estimate by accounting for variability across studies. To support this rationale, we have also cited [Dettori et al., 2022] in the revised manuscript, providing evidence that this approach is widely accepted in meta-analyses. We have updated the manuscript to reflect this clarification. (lines 228-233):

"Moreover, a random-effects model will be used to pool the calculated effect sizes across studies. This model was selected to account for variations between studies and individuals, making it appropriate for addressing the heterogeneity expected across studies (74). Unlike a fixed-effects model, which assumes a single common effect size, the random-effects model accommodates variations in effect sizes due to differences in study designs, populations, and interventions (74)"

While the ethics statement mentions that the study does not involve primary data collection and will assess ethical considerations of included studies (lines 209-211), authors might want to elaborate slightly on how this assessment will be done—whether specific frameworks will be used or if it will be handled case by case.

Thank you for your suggestion. We will assess the ethical considerations of included studies using a framework adapted from Hawker's quality assessment and risk of bias, focusing on Ethics (Were ethical issues, including confidentiality, sensitivity, and consent, properly addressed, and was necessary approval obtained?), Researcher-participant relationship (Was it adequately considered), Bias (Was the researcher reflexive or aware of their own bias?) (Lines 214-219)

While the manuscript mentions subgroup analyses to explore potential sources of heterogeneity (lines 219-220), consider indicating whether any visual representations of these subgroup analyses (e.g., stratified forest plots) will be included in the results.

Thank you for your feedback. Thank you for your suggestion. We will generate both pooled and stratified forest plots for the pre-specified and exploratory analyses (lines 257-258). Thus, we will visually represent the subgroup analyses and explore potential sources of heterogeneity.

Authors mentioned that sensitivity analyses will exclude studies with small sample sizes (lines 220-221), but it could help to briefly define what constitutes a "small" sample size in this context, or explain how you will determine the threshold.

Thank you for your suggestion. In response, we have clarified that a "small" sample size in the context of our sensitivity analyses will be defined as studies with fewer than 30 patients per group. This threshold aligns with common practices in meta-analyses. (Lines 258-261)

While authors do mention subgroup analyses by study design (lines 219-220), it might be helpful to expand slightly on why different designs (e.g., RCTs vs. quasi-experimental) could affect the results or introduce biases, just to emphasize the rationale for this analysis.

Thank you for this valuable suggestion. We acknowledge that different study designs, such as RCTs and quasi-experimental studies, may introduce varying levels of bias. RCTs are often regarded as the gold standard in clinical research due to their randomization process, which minimizes selection bias. Therefore, we will give more weight to RCTs in our analyses (lines 226-228). This approach is consistent with established guidelines that emphasize the higher methodological rigor of RCTs in comparison to quasi-experimental studies (Higgins et al., 2019: https://training.cochrane.org/handbook).

Reviewer #4

Can you explicitly state what your reported measures of effect will be (e.g., Cohen's d, Hedges' g etc) as to your primary research question?

Thank you for your comment. For our primary analysis, we will report Hedges' g as the measure of effect size. Hedges' g is chosen because it adjusts for potential bias in studies with small sample sizes, providing a more accurate estimate in these cases. While Cohen's d is widely used and effective for larger sample sizes, Hedges' g is preferred here due to the possibility of including smaller studies in our meta-analysis. We believe this will enhance the precision of our effect size estimates. The manuscript has been updated to reflect this (lines 224-226).

It is important to specify timepoints for the outcomes of interest from the DSM-5 outcome measures. For example, are you interested in measurements taken immediately, to 10-minutes post-intervention? And why? Please explicitly state this.

Thank you for your comment. To address variability in follow-up times across studies, we will categorize follow-up periods into short-term (0-6 months), medium-term (6-12 months), and long-term (>12 months) intervals for the outcomes of interest from the DSM-5 measures (Lines 245-253):

"To address variability in follow-up times across studies, we will categorize follow-up periods into short-term (0-6 months), medium-term (6-12 months), and long-term (>12 months) intervals for the outcomes of interest from the DSM-5 measures. These time categories are based on current evidence from behavioral intervention

studies, which indicate that different follow-up durations reflect distinct phases of intervention effectiveness and sustainability (75,76). This approach will allow us to assess both the immediate, short-term effects of XR-CBT interventions—capturing early symptom reduction and treatment adherence—as well as the long-term impacts, which are crucial for understanding sustained efficacy (77)."

As part of your synthesis plan, I recommend including a plan to perform an apriori meta-regression analysis on specific outcomes from the DSM-5 outcome measures relevant to mental health symptoms, especially if the number of studies is at minimum 10, included in the meta-analysis. I would also recommend you do a post-hoc exploratory meta-regression analysis on factors such as age, gender, and time since diagnosis (as these factors may be associated with symptoms associated with mental disorders).

Thank you for your valuable suggestion. We will include an a priori plan to perform a meta-regression analysis on DSM-5 outcomes relevant to mental health symptoms if at least 10 studies are included in the meta-analysis. This will allow us to explore potential associations between study characteristics and effect sizes. Additionally, we will conduct a post-hoc exploratory meta-regression to assess how factors such as age, gender, and time since diagnosis may influence mental health symptoms. These meta-regressions will help identify possible moderators of the intervention effects, further enhancing our synthesis of the evidence. The manuscript has been updated to reflect this plan (lines 233-237, lines 253-257):

"Additionally, we will conduct an a priori meta-regression within the random-effects framework to explore how study-level characteristics (e.g., sample size, study quality) may influence effect sizes and address heterogeneity across studies. This analysis will focus on specific outcomes from DSM-5 measures related to mental disorder symptoms, provided that at least 10 studies are included."

"Additionally, we will conduct a post-hoc exploratory meta-regression to assess the association between factors such as age, gender, and time since diagnosis and the severity of symptoms associated with mental disorders. These meta-regressions will help identify potential moderators of intervention effects, enhancing our understanding of the variability in study outcomes."

The hypothesis needs a minor revision to, "XR-CBT shows a greater effect on symptoms associated with mental disorders in children and youth compared to traditional therapies and the control condition (e.g., no treatment) at post-intervention".

Thank you for your feedback. The original hypothesis was, "XR-CBT shows a greater effect on mental disorders compared to traditional therapies and the control condition (e.g., no treatment) at post-intervention." We appreciate your suggestion to specify the focus on symptoms associated with mental disorders in children and youth. We recognize that your suggested revision adds clarity and aligns well with the study's scope. Therefore, we have revised the hypothesis to: "XR-CBT shows a greater effect on symptoms associated with mental disorders in children and youth compared to traditional therapies and the control condition (e.g., no treatment) at post-intervention." (lines 141-144)
