# Supplementary material for: The impact of extended reality cognitive behavioral therapy on mental disorders among children and youth: A systematic review and meta-analysis protocol
Source: PLoS One. 2025 Mar 6;20(3):e0315313. doi: 10.1371/journal.pone.0315313 (PMC11884679; doi:10.1371/journal.pone.0315313)
Supplement: Attachment — Submitted filename: Reviewer responses 3rd resubmission.pdf [file pone.0315313.s007.pdf]

Editor: Mohammad Jamil Rababa

1. Please review your reference list to ensure that it is complete and correct. If you have cited papers that have been retracted, please include the rationale for doing so in the manuscript text, or remove these references and replace them with relevant current references. Any changes to the reference list should be mentioned in the rebuttal letter that accompanies your revised manuscript. If you need to cite a retracted article, indicate the article's retracted status in the References list and also include a citation and full reference for the retraction notice.

   Thank you for your comment. We have carefully reviewed and confirmed that all references in our manuscript are complete and correct. Three references were updated to strengthen the alignment with our manuscript's content. Additionally, we verified that no retracted articles are included in our reference list.

Reviewer #2: Dear Authors,

1. While you provided valuable information on the adapted framework based on Hawker's quality assessment, it may be beneficial to briefly explain why this framework is especially suitable for assessing studies on XR-CBT in children and youth, enhancing clarity for readers.

   Thank you for your comment and the opportunity to provide clarity in our data extraction process. The Cochrane risk-of-bias tool is our primary quality appraisal tool, and it will be employed to evaluate bias risk for each study included in this systematic review and meta-analysis. The Cochrane risk-of-bias tool will examine potential biases related to randomization, allocation concealment, blinding, incomplete outcome data, and selective reporting of outcomes (lines 204-207).

   In response to comments/suggestions of another reviewer to elaborate on our ethics assessment, we used questions derived from the Hawker's assessment to guide our ethics evaluation, which Hawker's does more comprehensively than Cochrane risk-of-bias tool (lines 212-218). We created our questions based on the following specific questions from Hawker's quality appraisal: Were ethical aspects, including required approvals, confidentiality, sensitivity, and informed consent, comprehensively addressed? Additionally, was the interaction between researchers and participants thoughtfully considered?

   Hawker's framework uniquely supports evaluating ethical considerations such as the appropriateness of the consent process, the protection of participants' privacy, and the safeguarding of participants from any potential risks associated with XR technology—factors that are particularly pertinent for studies on emerging interventions like XR-CBT among young participants. Hawker's was not used as the main quality appraisal framework, but rather one of the components was used to supplement and enhance our primary quality appraisal.

We have now added this revision in the manuscript to the data screening and extraction section (lines 218-223): "Hawker's framework was selected as it uniquely supports the evaluation of ethical considerations, including the appropriateness of the consent process, the protection of participant privacy, and the safeguarding against potential risks associated with XR-CBT interventions (52). These aspects are particularly relevant for studies involving XR-CBT, given the emerging nature of these interventions (71,72) and the involvement of vulnerable populations such as youth and children (73–76)."

2. The addition explaining the rationale for analyzing different study designs is helpful; still, I believe that a brief note on specific biases that quasi-experimental studies might introduce (such as selection or performance bias) could further reinforce your rationale for prioritizing RCTs.

   Thank you for your comment and for highlighting the importance of addressing specific biases associated with the different study designs in our meta-analysis. To strengthen our rationale for prioritizing randomized controlled trials (RCTs) in this review, we have added a note on potential biases introduced by quasi-experimental studies, including selection bias, which may arise due to the lack of random assignment, and performance bias, which can result from inconsistent intervention delivery across non-randomized groups. By prioritizing RCTs, we aim to minimize these biases and ensure more robust conclusions on the efficacy of XR-CBT interventions.

   We have now added these revisions to the manuscript (lines 232-235): "In contrast, quasi-experimental studies can introduce potential biases, such as selection bias due to the lack of random assignment (81), and performance bias due to inconsistent intervention delivery across non-randomized study groups (82)."
