# Supplementary material for: The impact of extended reality cognitive behavioral therapy on mental disorders among children and youth: A systematic review and meta-analysis protocol
Source: PLoS One. 2025 Mar 6;20(3):e0315313. doi: 10.1371/journal.pone.0315313 (PMC11884679; doi:10.1371/journal.pone.0315313)
Supplement: Attachment — Submitted filename: Responsetoreviewers.docx [file pone.0315313.s005.docx]

**Administrative**

1. Please ensure that your manuscript meets PLOS ONE's style requirements, including those for file naming.
2. Thank you for stating the following financial disclosure: “Canada Research Chairs”
   a. Please state what role the funders took in the study. If the funders had no role, please state: "The funders had no role in study design, data collection and analysis, decision to publish, or preparation of the manuscript.
3. Please include this amended Role of Funder statement in your cover letter; we will change the online submission form on your behalf
4. We note that you have indicated that there are restrictions to data sharing for this study. PLOS only allows data to be available upon request if there are legal or ethical restrictions on sharing data publicly.
5. Please amend either the abstract on the online submission form (via Edit Submission) or the abstract in the manuscript so that they are identical
6. Please include captions for your Supporting Information files at the end of your manuscript, and update any in-text citations to match accordingly.

**Overall feedback**

1. Does the manuscript provide a valid rationale for the proposed study, with clearly identified and justified research questions? The research question outlined is expected to address a valid academic problem or topic and contribute to the base of knowledge in the field.

- Reviewer #1: Partly
- Reviewer #2: Yes
- Reviewer #3: Yes
- Reviewer #4: Partly

2. Is the protocol technically sound and planned in a manner that will lead to a meaningful outcome and allow testing the stated hypotheses? The manuscript should describe the methods in sufficient detail to prevent undisclosed flexibility in the experimental procedure or analysis pipeline, including sufficient outcome-neutral conditions (e.g. necessary controls, absence of floor or ceiling effects) to test the proposed hypotheses and a statistical power analysis where applicable. As there may be aspects of the methodology and analysis which can only be refined once the work is undertaken, authors should outline potential assumptions and explicitly describe what aspects of the proposed analysis, if any, are exploratory.

- Reviewer #1: Yes
- Reviewer #2: Yes
- Reviewer #3: Partly
- Reviewer #4: No

3. Is the methodology feasible and described in sufficient detail to allow the work to be replicable? Descriptions of methods and materials in the protocol should be reported in sufficient detail for another researcher to reproduce all experiments and analysis. The protocol should describe the appropriate controls, sample size calculations, and replication needed to ensure that the data are robust and reproducible.

- Reviewer #1: Yes
- Reviewer #2: Yes
- Reviewer #3: Yes
- Reviewer #4: No

4. Have the authors described where all data underlying the findings will be made available when the study is complete? The [PLOS Data policy](https://c05y1x9s.r.us-east-2.awstrack.me/L0/https:%2F%2Fjournals.plos.org%2Fplosone%2Fs%2Fmaterials-and-software-sharing/1/010f0191de40d659-7ed04be8-6b9d-4cc4-8118-75724af1fd5b-000000/QHSv909ixFM_IRiQ4nu_i6OS5qg=176) requires authors to make all data underlying the findings described in their manuscript fully available without restriction, with rare exception, at the time of publication. The data should be provided as part of the manuscript or its supporting information, or deposited to a public repository. For example, in addition to summary statistics, the data points behind means, medians and variance measures should be available. If there are restrictions on publicly sharing data—e.g. participant privacy or use of data from a third party—those must be specified.

- Reviewer #1: No
- Reviewer #2: Yes
- Reviewer #3: Yes
- Reviewer #4: No

5. Is the manuscript presented in an intelligible fashion and written in standard English? PLOS ONE does not copyedit accepted manuscripts, so the language in submitted articles must be clear, correct, and unambiguous. Any typographical or grammatical errors should be corrected at revision, so please note any specific errors here.

- Reviewer #1: Yes
- Reviewer #2: Yes
- Reviewer #3: Yes
- Reviewer #4: Yes

**Reviewer #1**

1. The manuscript provides a strong rationale for exploring XR-CBT and clearly outlines the research questions. However, further elaboration on how XR-CBT uniquely addresses the mental health needs of children and youth compared to traditional CBT would strengthen the justification for the study.

Thank you for your suggestion. We have now added more content to describe the limitations of traditional CBT and how XR-CBT offers benefits for addressing them, with specific findings from previous literature: “Moreover, previous evidence has shown XR-CBT interventions to be more acceptable and lead to higher satisfaction levels compared to traditional therapeutic approaches (42,43). Among patients with specific phobias, the refusal rate for VRET was 3%, substantially lower than the 27% refusal rate observed with in-vivo exposure (43). In a study examining soldier attitudes on technology-based approaches in mental health care treatment, 19% percent of individuals who indicated they were unwilling to speak with a counsellor in person reported a willingness to use VR approaches for accessing mental health care, indicating that VR may help overcome certain barriers to treatment (44).” Lines 96-103

2. The methodology is robust and follows PRISMA-P guidelines, ensuring a structured approach to the systematic review. However, providing additional details on the data synthesis method (e.g., narrative synthesis or meta-analysis) would enhance the transparency of the planned analysis.

Thank you for your feedback. We have now added a section on the data synthesis methods (lines 213 to 220): “All data analysis and statistical modeling will be conducted using RStudio (63). We will extract post-intervention means, standard deviations (SDs), and 95% confidence intervals (CIs) from each study, and we will calculate standardized mean differences (SMDs) for the effect size. A random-effects model will be used to pool the calculated effect sizes across studies. Forest plots will be generated to visually represent the pooled effect sizes with corresponding confidence intervals. Heterogeneity will be assessed using the I² statistic and Cochran’s Q test. Subgroup analysis will be performed to examine differences by study design, intervention type, and participant characteristics.”.

3. Consider including a brief discussion on any exploratory analysis planned or how potential methodological challenges will be managed.

Thank you for your suggestion. We added more details regarding our approach to our planned meta-analysis above (lines 213 to 220). We have also added content on how we will address potential methodological challenges (lines 220 to 225): “Sensitivity analysis will be conducted by excluding studies with small sample sizes or high risk of bias. Publication bias will be assessed using funnel plots and statistically tested using Egger’s regression test. When available, intention-to-treat (ITT) data will be used for the primary analysis. Additionally, any missing data or incomplete reporting will be addressed by contacting the study authors for clarification.”.

4. The protocol currently does not specify where the data underlying the findings will be made available upon completion of the review. Please include a statement detailing the data-sharing plan, such as depositing the data in a public repository, to comply with PLOS ONE’s data policy. This will ensure transparency and accessibility of the review's findings.

A statement on data availability has been included in our protocol (Lines 253 to 256): Post-intervention data used in this review, including standard deviations, means, 95% confidence intervals, and effect sizes, will be made publicly accessible upon publication. These data will be deposited in a publicly available repository to ensure transparency and replicability of the findings. However, we will not be collecting any primary data as a part of this review.

5. Only minor language polishing is needed to improve readability and flow.

Thank you for your feedback. We have proofread the manuscript to improve readability and flow.

6. Consider adding a brief theoretical framework that connects XR technologies with CBT principles to strengthen the conceptual basis of the review.

Thank you for your feedback, we understand the importance of connecting XR-CBT interventions with core CBT principles to strengthen the conceptual basis of our systematic review.

We have now defined the core principles of CBT: “CBT is based on the cognitive theory that negative thought patterns lead to emotional distress and behavioral issues (14-16). Through structured therapy sessions, CBT enables individuals to recognize these maladaptive thoughts, challenge their accuracy, and modify behaviors (17)”. (Lines 67 to 70)

Thereafter, we described how XR technologies can build on these theoretical principles (Lines 90 to 96): “These XR interventions builds on core CBT principles by creating immersive environments that incorporate the immersive nature of XR to create realistic scenarios where patients can practice coping strategies, desensitize themselves to anxiety-provoking stimuli, and receive real-time feedback from their therapists (38-41). The immersive nature of XR-CBT supports the cognitive-behavioral process of thought-behavior-emotion integration by directly engaging the patient in realistic scenarios where learned skills can be applied, which can strengthen therapeutic outcomes (26,34).”

7. Expanding on the future implications and potential challenges of XR-CBT (e.g., ethical considerations, cost, and accessibility issues) would provide a more comprehensive outlook on its practical application.

Thank you for your feedback. We have added the potential challenges of XR-CBT in the Introduction (Lines 112 to 118): “However, current evidence on the benefits of VR lacks consensus – some studies report benefits such as enhanced cognitive, motivational, emotional, and social development, while others highlight risks including addiction, anxiety, emotional distress, sleep problems, cyber sickness, and obesity (51). Given the double-edged nature of XR interventions for mental health, the high prevalence of mental disorders among children and youth (9,10,12), and the limitations of traditional CBT (25–30), it is crucial to investigate the efficacy of XR-CBT for this demographic.”.

**Reviewer #2:**

1. The introduction could benefit from a more extensive literature review that discusses existing studies on XR-CBT, particularly those involving children and youth. This would help establish a stronger foundation for the need for this systematic review.

Thank you for your feedback and the opportunity to expand on the background information regarding the use of XR therapies among children and youth. Our revised introduction now provides a more comprehensive literature review, presenting evidence of how XR therapies are utilized across various mental health issues in this population. For instance, we have now added (Lines 106 to 112): “The advancement of XR in mental health care has been applied to treat various mental disorders among children and youth. For instance, VR therapy interventions have been used to address anxiety disorders among children and youth, particularly for specific phobias where traditional exposure therapy is difficult to conduct (48). Moreover, current evidence suggests that VR interventions are an effective and well-accepted treatment approach for addressing psychological distress among adolescents (49) . For instance, within hospital settings, VR has been used as an engaging therapeutic method to alleviate pain and anxiety among in-patient adolescents (50).”.

Additionally, we have cited and described evidence underscoring the potential safety and ethical concerns associated with these interventions (lines 112 to 118). This balanced perspective provides a stronger foundation for our review, demonstrating the critical need for further exploration into both the benefits and challenges of XR therapies for children and youth.

Overall, we have added 10 additional references citing existing studies on XR-CBT to enhance our literature review.

2. While the paper mentions the databases to be used for the literature search, it lacks specific details about the search strategy (e.g., keywords, inclusion/exclusion criteria). I would ask the authors to provide this information to enhance the reproducibility of the review.

Thank you for your suggestion to include specific details about the search strategy, such as keywords and inclusion/exclusion criteria. Table 1 (Line 173) outlines the inclusion and exclusion criteria, and Table 2 (Line 191), details the key concepts and search terms used in our literature search. Nevertheless, we have now uploaded our full search strategy as a supplementary file.

3. The paper should address potential biases in the selection of studies, such as publication bias or language bias. Including a plan for assessing the risk of bias in the included studies would strengthen the review's credibility.

Thank you for your feedback. We would like to clarify that language bias has been addressed in the limitations section of our protocol, highlighting that studies included in our systematic review are limited to manuscripts published in English (lines 236 to 238). Moreover, we have included a statement that indicates how the Cochrane risk-of-bias tool will be used to assess the risk of bias for individual studies included in the systematic review (lines 201 to 204).

We have also added to the Strengths and Limitations: “This review will include all studies on XR-CBT on mental health outcomes among youth and children, regardless of their results.”(Lines 243 to 246). Moreover, we have now added to the Methods: “publication bias will be assessed using funnel plots and statistically tested using Egger’s regression test.” (lines 221 to 222).

4. Although the paper mentions the need for an ethics statement, it does not elaborate on how ethical considerations will be handled, particularly given the vulnerable population involved (children and youth).

Thank you for highlighting the importance of ethical considerations, particularly when discussing vulnerable populations such as children and youth. We have now added an ethics statement to our data screening and extraction section: “This study does not involve the collection of primary data and therefore does not require ethics approval. However, we will assess and report on the ethical considerations related to the studies included, such as informed consent and confidentiality, where applicable.” (Lines 209 to 211)

5. The authors state that no datasets will be generated or analyzed during the current study. However, they should clarify how they plan to share any findings or insights from the review, as transparency in data availability is increasingly important in research.

A statement on data availability has been included in our protocol. Specifically, we will ensure that post-intervention means, standard deviations, 95% confidence intervals, and standardized mean differences for the effect size of each study will be made publicly available in our manuscript. However, we will not be collecting any primary data as a part of this review. (Lines 253 to 256)

6. The review should specify how the impact of XR-CBT will be measured. Authors should better define clear metrics or outcomes for evaluating mental health outcomes will enhance the study's applicability and relevance.

Thank you for your valuable feedback. We will perform a meta-analysis to quantitatively synthesize the results from the included studies. We have now added a section on the data synthesis methods (lines 213 to 220): “All data analysis and statistical modeling will be conducted using RStudio (63). We will extract post-intervention means, standard deviations (SDs), and 95% confidence intervals (CIs) from each study, and we will calculate standardized mean differences (SMDs) for the effect size. A random-effects model will be used to pool the calculated effect sizes across studies. Forest plots will be generated to visually represent the pooled effect sizes with corresponding confidence intervals. Heterogeneity will be assessed using the I² statistic and Cochran’s Q test, with 25%, 50%, and 75% indicating low, moderate, and high levels. Subgroup analyses will be performed to examine differences by study design, intervention type, and participant characteristics.”

Moreover, we have now added a new section in the Methods to describe the mental health outcomes assessed in this study: “This review broadly considers mental disorders as defined by the Diagnostic and Statistical Manual of Mental Disorders, Fifth Edition (DSM-5), which provides the standard classification of mental disorders used by mental health professionals (55). The DSM-5 outlines specific criteria for diagnosing mental health conditions, including disorders related to mood (such as depression), anxiety (such as generalized anxiety disorder, phobias, and social anxiety), and other conditions like ADHD, obsessive-compulsive disorder (OCD), and post-traumatic stress disorder (PTSD). Each disorder is characterized by a unique set of symptoms and diagnostic criteria, and the review takes into account the diversity of these conditions when examining the potential applications of XR-CBT across different mental health issues.” (Lines 140 to 148)

**Reviewer #3**

1. The Manuscript needs careful English proofreading because there are some shortcomings. For instance, the article “the” is sometimes missing in front of nouns, the message in some paragraphs is not clear enough. It looks like the first part was written by one author with a greater command of the English language, and the rest of the paper was written by someone else. The numerous grammar errors made this a difficult paper to read. It was strange to see the authors refer to tables that were not submitted. I was unable to find any supplementary material to the submission, so I think this was truly omitted by the authors. Please read the manuscript carefully.

Thank you for your feedback and for highlighting the areas where the manuscript could be improved. We have carefully proofread the document prior to submission, but we will conduct another thorough review to ensure clarity and correct any grammatical issues, including the use of articles and any other shortcomings. We have also ensured that all necessary supplementary files are correctly included.

2. The abstract must contain the main purpose of the paper, the research method used in the research and the main contributions.

Thank you for your feedback and the opportunity to improve the clarity in our abstract.

1) We added the purpose of the paper on to lines 36 to 38: “Objectives: This systematic review and meta-analysis protocol aims to outline the methodology for conducting a systematic review and meta-analysis to evaluate the impact of XR-CBT on reducing symptoms of mental disorders among children and youth.”
2) We added details regarding the research methods to lines 39 to 50: “Methods: A comprehensive literature search will be conducted using five databases: PsycINFO, PubMed, EMBASE, Scopus, and Web of Science. The search strategy will include terms related to virtual reality, augmented reality, mixed reality, cognitive behavioral therapy, exposure therapy, youth, children, and mental health. Two independent reviewers will evaluate the search results from the five selected databases based on the pre-determined search strategy. Moreover, a meta-analysis will be conducted to assess the efficacy of XR-CBT in reducing symptoms of mental disorders among children and youth. Post-intervention standard deviations and means will be extracted, and 95% confidence intervals will be calculated for standardized mean differences. Heterogeneity will be assessed using the I² statistic and Cochran’s Q test.”
3) Finally, we added the main contributions to lines 51 to 54: “Conclusions: This protocol establishes a clear methodology for systematically assessing the impact of XR-CBT on mental disorders among children and youth, addressing a gap in existing research and contributing to solutions for the escalating mental health crisis in this population.”
3. The last section must contains Conclusions and Policy Implications

Thank you for your feedback. As part of our conclusions section, we have now added policy implications of our research, highlighting the potential of incorporating XR-CBT interventions into existing mental health care frameworks: “Additionally, findings from this review will not only contribute to the existing body of literature but will also offer evidence-based recommendations for policy-makers and mental health practitioners on how to effectively implement XR-CBT interventions. This includes considerations for integrating these technologies into existing mental health care frameworks, ensuring accessibility, and addressing the unique needs of young patients.” (Lines 242 to 247).

4. It would be very useful to add the "Introduction" section and the purpose, objectives and hypothesis of the research. I consider that a weak point of the paper is that the authors did not show the novelty of the paper compared to other works. That is why, I consider that the introduction should specify the novelty of the paper compared to other papers published in this area.

Thank you for your feedback. We have now clarified our purpose, objectives, and hypothesis in our introduction.

The purpose of our research has been added to lines 119 to 124. We emphasize the need to systematically review evidence on XR-CBT on mental disorders among children and youth based on the current gap in research: “Currently, no systematic reviews have explored the impact of XR-CBT on mental disorders among children and youth. Prior reviews have examined the effectiveness of XR-CBT therapy interventions; however, they have primarily focused on general populations rather than children and youth (24,34,35). While reviews on XR interventions among children and youth exist, they have focused on specific mental health issues, such as anxiety (36) or schizophrenia (37), rather than evaluating the broader applications of XR-CBT".

The objective of our research has been added to lines 124 to 127: “Thus, this review aims to quantitatively assess the impact of XR-CBT interventions on mental disorders among children and youth. Moreover, this review will also generate recommendations for future development, implementation, and evaluation of XR-CBT that aims to treat youth and child mental health.”

Lastly, the hypothesis of our research has been added to lines 134 to 136: XR-CBT shows a greater effect on mental disorders compared to traditional therapies and the control condition (e.g., no treatment) at post-intervention.

5. The Literature Review section cannot be missing from the paper.

Thank you for your feedback. Our revised introduction now provides a more comprehensive literature review, presenting evidence of how XR therapies are utilized across various mental health issues in this population.

For instance, we have now added (Lines 106 to 112): “The advancement of XR in mental health care has been applied to treat various mental disorders among children and youth. For instance, VR therapy interventions have been used to address anxiety disorders among children and youth, particularly for specific phobias where traditional exposure therapy is difficult to conduct (48). Moreover, current evidence suggests that VR interventions are an effective and well-accepted treatment approach for addressing psychological distress among adolescents (49). For instance, within hospital settings, VR has been used as an engaging therapeutic method to alleviate pain and anxiety among in-patient adolescents (50)”

Additionally, we have cited and described evidence underscoring the potential safety and ethical concerns associated with these interventions (lines 112 to 118). This balanced perspective provides a stronger foundation for our review, demonstrating the critical need for further exploration into both the benefits and challenges of XR therapies for children and youth.

6. Authors must present the software used in data processing (STATA, Eviews, etc)

Thank you for your feedback. To clarify, Covidence software will be used in this review for screening and management of studies (Lines 194 to 195, and Line 204). For our meta-analysis, RStudio will be used for data analysis and statistical modeling, including pooling effect sizes, and conducting subgroup analysis (Line 213)

7. In the same time, the authors must present a minimum an empiric model.

Thank you for your valuable feedback. In response to your suggestion, we will conduct a meta-analysis to quantitatively assess the efficacy of XR-CBT in reducing symptoms of DSM-5-defined mental disorders among children and youth. We have added to the Methods (lines 215 to 220): “A random-effects model will be used to pool the calculated effect sizes across studies. Forest plots will be generated to visually represent the pooled effect sizes with corresponding confidence intervals. Heterogeneity will be assessed using the I² statistic and Cochran’s Q test [....].

Additionally, we have enhanced the Introduction by incorporating a comprehensive literature review to provide context for the empirical model, outlining key studies and findings in the field (lines 96 to 105, lines 106 to 118). This will offer a clear theoretical foundation and justify the rationale for conducting the systematic review and meta-analysis.

8. I recommend the authors to refer to other recent works indexed in Web of Science, because the papers cited works is not enough for a scientific paper. In my opinion, the authors must cite other papers regarding this subject.

Thank you for your feedback and the opportunity to cite additional papers on XR-CBT. We have now added 10 references to our introduction, presenting a more extensive literature review of past evidence on XR-CBT. Our revised introduction now provides a more comprehensive literature review to add articles on utilized XR-CBT among the children and youth population, including works indexed in the Web of Science database. Specifically, we have presented evidence of how XR therapies are utilized across various mental health issues in this population. Additionally, we have underscored the potential safety and ethical concerns associated with these interventions. In the forthcoming meta-analysis, we will extract studies from various databases, including PubMed, Embase, Web of Science, Scopus, and PsycInfo, to ensure a diverse range of studies are included.

9. The conclusions at the end of the paper should be expanded showing the economic policy implications of the research results. In conclusion, the article should be improve. It should also be enhanced with a review of the literature adequate to the subject and a broader interpretation and commentary of the research results.

In response to a different reviewer’s comments, we have decided to narrow our research questions to focus solely on the efficacy of XR-CBT in reducing symptoms of mental disorders among children and youth. The objectives of our meta-analysis will be: “To quantitatively assess the impact of XR-CBT interventions on mental disorders among children and youth. Moreover, this review will also generate recommendations for future development, implementation, and evaluation of XR-CBT that aims to treat youth and child mental health.” (lines 124 to 127)

As a result, we will not be including an analysis of economic policy within the scope of this review. We have addressed the broader policy implications in the discussion section of the final study, contextualizing our findings within healthcare systems and policy impacts: “findings from this review will not only contribute to the existing body of literature but will also offer evidence-based recommendations for policy-makers and mental health practitioners on how to effectively implement XR-CBT interventions. This includes considerations for integrating these technologies into existing mental health care frameworks, ensuring accessibility, and addressing the unique needs of young patients”. (Lines 242 to 247)

Moreover, we will discuss the potential economic policy implications in the discussion section of the final study, where we will contextualize our findings in relation to broader health care and policy impacts.

R**eviewer #4:**

1. I recommend a major re-conceptualisation and rewrite regarding the specifics of the review. In particular, I am concerned how particular constructs are defined (or lack thereof), and what outcome measurements are of interest for analysis.

Thank you for your feedback. Our outcome of interest is mental disorders as defined by the DSM 5. We followed the approach taken by previous studies assessing the impact of VR on mental disorders.

- <https://www.ncbi.nlm.nih.gov/pmc/articles/PMC7468638/>
- <https://www.sciencedirect.com/science/article/pii/S0272735822000988#s0010>

We have now added an outcomes section to the Methods (lines 140 to 148): “The review broadly considers mental disorders as defined by the **Diagnostic and Statistical Manual of Mental Disorders, Fifth Edition (DSM-5)**, which provides the standard classification of mental disorders used by mental health professionals. The DSM-5 outlines specific criteria for diagnosing mental health conditions, including disorders related to mood (such as **depression**), anxiety (such as **generalized anxiety disorder, phobias, and social anxiety**), and other conditions like **ADHD**, **obsessive-compulsive disorder (OCD)**, and **post-traumatic stress disorder (PTSD)**. Each disorder is characterized by a unique set of symptoms and diagnostic criteria, and the review takes into account the diversity of these conditions when examining the potential applications of XR-CBT across different mental health issues.”

2. I recommend the authors follow pre-registration guidance at Prospero and/or OSF to tightly define the review's scope before proceeding any further. Currently, the protocol is close to being broad enough to perform a scoping review; however as a systematic review and meta-analysis the study design needs major revisions before being accepted as a published report. I have provided comments within the text for areas of revision.

Thank you for your feedback. Following our registration on PROSPERO, we refined our eligibility criteria to align with the registration requirements (Lines 137 to 166), using the PICO framework to ensure clarity and focus. This approach helped guide the development of our systematic review and meta-analysis, ensuring consistency with the registered protocol.

3. Does “youth” refer to minors, young adults? In other words, it would be worth specifying the age demographic here. Among youth “age = 18–31” as an example. Also, please provide a citation where possible. Here is your target group for analysis? I would update the previous sentence: “among youth (e.g., adolescent ages 10–19).

Thank you for your feedback. Our manuscript focuses on the child and youth population. The World Health Organization (WHO) classifies a “child” as any person under the age of 18, and the WHO and United Nations classifies “youth” as any person within the 15-24 year age group. Moreover, the WHO defines “adolescents” as any person in the 10-19 years age group. Many of these definitions have overlapping age ranges, which is consistent with a previous systematic review on mental health that have used similar age cohorts, such as ages 2-24:

- <https://capmh.biomedcentral.com/articles/10.1186/s13034-023-00629-4>)

Thus, our study will focus on the child and youth populations (ages 24 years and younger). We have now revised our manuscript to provide citations regarding these age ranges to give more clarification, which can be found in the eligibility criteria (line 138). Moreover, our definition of “children and youth” has been defined in the methods section of our manuscript.

4. A good question for meta-analysis – but you must clarify the mental health symptoms of interest. E.g., XR-CBT’s effects on depression severity? On anxiety severity? Etc.

Thank you for your feedback. As mentioned in response to question 1, our meta-analysis will focus on the primary outcome of mental disorders defined by the **Diagnostic and Statistical Manual of Mental Disorders, Fifth Edition (DSM-5)**, which provides the standard classification of mental disorders used by mental health professionals. We have provided a definition of our primary outcome in our manuscript in lines 140 to 148.

5. Can you elaborate/clarify in the introduction, regarding customisation and personalisation of XR-CBT interventions? Can you clarify “digital mental health platforms” in the introduction? I would remove this RQ entirely, and perhaps discuss this as background or narratively within the review.

Thank you for your feedback. In response to your comment, we have decided to narrow our research questions to focus solely on the efficacy of XR-CBT in reducing symptoms of mental	disorders among children and youth. The objectives of our meta-analysis will be: “To quantitatively assess the impact of XR-CBT interventions on mental disorders among children and youth. Moreover, this review will also generate recommendations for future development, implementation, and evaluation of XR-CBT that aims to treat youth and child mental health.” (Lines 124 to 127)

Thus, we have also removed our section of digital mental health platforms and customization and personalization in our introduction as well, clarifying the focus of	our research.

6. What specific variables constitute “mental health outcomes”? In other words, how will you examine mental health as a construct in the review? Will it be based on questionnaire outcomes, self-report outcomes? For example, you could be more precise here and state mental health outcomes such as reduced anxiety, depression, stress as indicated via several questionnaire indicies e.g., Perceived Stress Scale (PSS) etc. The crucial point here, is that you describe how mental health outcomes will be measured, what data creates this construct and report this precisely.

Thank you for your feedback. We will examine mental disorders based on validated scales that capture constructs such as anxiety, depression, and stress-related disorders. Specifically, we will include outcomes measured through different instruments, such as the Perceived Stress Scale (PSS), the Beck Depression Inventory (BDI), and the Generalized Anxiety Disorder scale (GAD-7) (Lines 149 to 151), which has been done by a previous study (https://bmjopen.bmj.com/content/13/7/e073660 )

These scales will allow us to assess the mean differences in mental disorders across studies, ensuring consistency and precision in how mental disorders are measured throughout the review.

7. Eligibility criteria: This is somewhat disconnected from the population described in the Introduction; I recommend revising the introduction to describe young adults more generally.

Thank you for your feedback. As mentioned in response to question 3, we have now defined our definition of youth (ages 24 years and younger) in the Introduction and cited previous evidence that corroborates this age range. This is also now reflected in our eligibility criteria.

8. Would this comparator/control group encompass traditional CBT (i.e., not augmented with XR/VR/AR)? Please specify this, and also provide a stronger rationale, how the effects would be different across the groups.

Thank you for your feedback and the opportunity to clarify our intervention and control groups to be analyzed in our meta-analysis. We have now revised our inclusion and exclusion criteria for more clarity on what our study will be comparing. Specifically, the alternatives against which the intervention (XR-CBT) will be compared in this review include traditional therapy, stand-alone cognitive behavioral therapy (CBT), and/or a control group, such as no treatment or placebo. Below is a statement of our inclusion and exclusion criteria for our therapeutic interventions of interest:

- Inclusion criteria: studies that will be included in the review must compare XR-CBT with traditional therapies, such as in-person therapy, or stand-alone CBT. Additionally, studies that include a control group, such as those receiving no treatment, placebo, or standard care, will also be eligible for inclusion. (Line 173)

- Exclusion criteria: studies that do not compare XR-CBT interventions with an alternative therapy or control will be excluded. (Line 173)

Moreover, our introduction now provides a stronger rationale for comparing XR-CBT interventions to traditional therapy, stand-alone cognitive behavioral therapy (CBT), and/or a control group, such as no treatment or placebo. We highlight unique advantages of XR-CBT compared to traditional approaches, such as high acceptability and satisfaction: “Moreover, previous evidence has shown XR-CBT interventions to be more acceptable and lead to higher satisfaction levels compared to traditional therapeutic approaches (34,35). Among patients with specific phobias, the refusal rate for VRET was 3%, substantially lower than the 27% refusal rate observed with in-vivo exposure (35). In a study examining soldier attitudes on technology-based approaches in mental health care treatment, 19% percent of individuals who indicated they were unwilling to speak with a counsellor in person reported a willingness to use VR approaches for accessing mental health care, indicating that VR may help overcome certain barriers to treatment” (Lines 96 to 103)

Moreover, we have also highlighted the utilization of XR-CBT among different mental disorders in children and youth: “VR therapy interventions have been used to address anxiety disorders among children and youth, particularly for specific phobias where traditional exposure therapy is difficult to conduct (40). Moreover, current evidence suggests that VR interventions are an effective and well-accepted treatment approach for addressing psychological distress among adolescents. Moreover, within hospital settings, VR has been used as an engaging therapeutic method to alleviate pain and anxiety among in-patient adolescents (50)” (Lines 107 to 112)

9. Can you clarify, does the exclusion criteria also include traditional CBT?

Thank you for your feedback. As mentioned in the answer to question 8, we are examining traditional CBT as a comparator only to XR-CBT as the intervention group (line 173)

10. Can you define what this construct is? “Mental wellbeing”

Thank you for your feedback. As mentioned before in a question above, we have now changed our primary outcome to mental disorders defined by the Diagnostic and Statistical Manual of Mental Disorders, Fifth Edition (DSM-5) (lines 140 to 148). Thus, we have removed all constructs related to mental wellbeing.

11. Do you mean experimental studies? What about observational studies? Randomised Controlled Trials? Be specific here what you will include.

Thank you for your feedback. We have now refined our inclusion and exclusion criteria for which type of studies to include in this systematic review and meta-analysis:

The review will only include experimental study designs such as randomized controlled trials (RCTs), quasi-experimental studies, and controlled clinical trials (CCTs) that examine the effects of XR-CBT interventions on mental disorders in children and youth.

Inclusion Criteria:

Studies with experimental designs, including RCTs, quasi-RCTs, and CCTs, that assess the efficacy of XR-CBT interventions in treating mental disorders.

Exclusion Criteria:

Observational studies, case studies, feasibility studies, developmental studies, or studies that do not employ experimental or quasi-experimental designs.

This revision is now reflected in the Methods. (Line 173)

12. Will you perform a narrative synthesis of the data? Will you attempt a meta-analysis, and which type of meta-analysis, (random-effects vs. fixed-effects model; I’d recommend random-effects because of the potential heterogenity of the intervention(s)) and why (provide a rationale)?

Thank you for your feedback. We will perform a meta-analysis to quantitatively synthesize the results from the included studies. We have now added a section on the data synthesis methods (lines 213 to 220) “All data analysis and statistical modeling will be conducted using RStudio (64). We will extract post-intervention means, standard deviations (SDs), and 95% confidence intervals (CIs) from each study, and we will calculate standardized mean differences (SMDs) for the effect size. A random-effects model will be used to pool the calculated effect sizes across studies. Forest plots will be generated to visually represent the pooled effect sizes with corresponding confidence intervals. Heterogeneity will be assessed using the I² statistic and Cochran’s Q test. Subgroup analysis will be performed to examine differences by study design, intervention type, and participant characteristics.”.

By aggregating and synthesizing data from various trials, we can provide a more robust and reliable estimate of the impact of XR-CBT on mental disorders among children and youth. This approach is particularly valuable as it enables us to determine the overall impact of XR-CBT while accounting for variations in study design, intervention methods, and populations. By focusing on effectiveness, the meta-analysis will help us draw clearer conclusions about the true therapeutic potential of XR-CBT.

13. What are your outcomes of interest? What constructs related to mental health are measured in CBT practices that can be reliably transferred to XR/VR-CBT therapies? Are you interested in depression severity or anxiety severity? Please be specific.

Thank you for your feedback. As mentioned in our response to question 1, we have now	refined our outcome to mental disorders as defined by the Diagnostic and Statistical Manual	of Mental Disorders, Fifth Edition (DSM-5) (added to lines 140 to 148).

14. Sensitivity analyses should be conducted to test the robustness of the results. For instance, analysis excluding studies with high risk of bias or focusing only on higher-quality studies can help determine how these factors influence overall findings.

Thank you for your feedback. We have now added sensitivity analysis to the data screening and extraction section of our protocol: “Sensitivity analysis will be conducted by excluding studies with small sample sizes or high risk of bias”. (Lines 220 to 221)

15. Regarding a meta-analysis, clarify which effect size measures will be used for different outcome types. E.g., for anxiety or depression severity scores, the authors could use standardised mean differences (SMD) or weighted mean differences (WMD).

Thank you for our feedback. We have now added the effect size measures that will be used for our primary outcome of mental disorders: “We will extract post-intervention means, standard deviations (SDs), and 95% confidence intervals (CIs) from each study, and we will calculate standardized mean differences (SMDs) for the effect size. A random-effects model will be used to pool the calculated effect sizes across studies.” we will calculate standardized mean differences (SMDs) to standardize effect sizes across different outcome measures. (Lines 213 to 215)

Moreover, we will perform subgroup analysis for different outcome types: “Subgroup analysis will be performed to examine differences by study design, intervention type, and participant characteristics”. (Lines 219 to 220)

16. Please specify how heterogeneity will be measured, such as through I² statistics or Cochran’s Q test. The authors should define thresholds for low, moderate, and high heterogeneity (e.g., I² values of 25%, 50%, and 75% respectively).

Thank you for your feedback. We have now added how heterogeneity will be measured in our meta-analysis in lines 218 to 219: “Heterogeneity will be assessed using the I² statistic and Cochran’s Q test, with 25%, 50%, and 75% indicating low, moderate, and high levels.”

17. In cases where the data are too heterogeneous for meta-analysis, a clear plan for narrative synthesis should be outlined. This could include qualitative comparisons between studies or descriptive analysis of study characteristics and outcomes.

Thank you for your feedback. We understand the importance of addressing heterogeneity in our studies. To address the heterogeneity of studies included in our systematic review and meta-analysis, we will conduct subgroup analyses to explore potential sources of variability (lines 219 to 220). Additionally, the use of a random-effects model will account for unexplained heterogeneity across studies (lines 213 to 215). Furthermore, sensitivity analyses will be performed by excluding outlier studies, which will provide further insight into the impact of heterogeneity on the overall findings (lines 220 to 221).
